# Epidemiological, Clinical, and Virological Investigation of the First Four Cases of Monkeypox in Cartagena during the 2022 Outbreak

**DOI:** 10.3390/pathogens12020159

**Published:** 2023-01-18

**Authors:** Steev Loyola, Mashiel Fernández-Ruiz, Doris Gómez-Camargo

**Affiliations:** 1 Grupo de Investigación UNIMOL, Facultad de Medicina, Universidad de Cartagena, Cartagena de Indias 130014, Colombia; 2 Doctorado en Medicina Tropical, Facultad de Medicina, Universidad de Cartagena, Cartagena de Indias 130014, Colombia

**Keywords:** Monkeypox, men having sex with men, outbreak, imported, Colombia, Cartagena de Indias

## Abstract

Since early May 2022, numerous cases of Monkeypox (Mpox) have been reported globally in non-endemic areas. However, despite numerous reports worldwide, the epidemiological and genomic information related to the 2022 multi-country outbreak remains scarce in South America. By late June 2022, the first Mpox cases were detected in Colombia. Cartagena is a Colombian Caribbean city with high domestic and international connectivity, and, therefore, is vulnerable to the introduction of the Monkeypox virus (MPXV). This report provides an in-depth description of the epidemiological, clinical, and virological characteristics of the first four cases detected in Cartagena including three cases with no history of recent travel and one imported case. Using various laboratory tools based on PCR, next-generation sequencing, and viral isolation and quantification methods, the MPXV clade IIB was detected and isolated. Importantly, infectious viral particles were identified in lesion swabs collected from all cases and in oropharyngeal swabs collected from two cases. Blood samples tested negative using PCR and isolation. In summary, our work contributes complete genomic, clinical, and epidemiological information that will be useful for a number of studies going forward, and it also documents local information that contributes to our understanding of Mpox at the local level.

## 1. Introduction

Monkeypox (Mpox) is a zoonotic human-transmitted disease caused by the Monkeypox virus (MPXV). The first human case was reported in the Democratic Republic of Congo in 1970 [1]. Since then, multiple cases and sporadic outbreaks in humans have been reported in several African cities largely caused by MPXV clade I (formerly known as the Central African clade) [2]. However, it was not until 2003 that the first cases and outbreaks were reported outside of Africa mostly caused by MPXV clade II (formerly known as the West African clade) [2]. Historically, infections caused by MPXV clade I were associated with a higher case fatality rate compared to those caused by MPXV clade II [2]. Contact with infected animals or fomites and travel to areas of confirmed transmission have been described as the main transmission mechanisms and factors associated with Mpox in areas without sustained historical human-to-human transmission [2,3]. Increased connectivity between endemic and non-endemic areas raises the risk of Mpox importation and spread [3,4].

Since early May 2022, numerous cases were reported mainly in multiple localities throughout the European region [5]. Given the substantial increase of MPXV infections globally, on 23 July, the World Health Organization declared the Mpox outbreak a Public Health Emergency of International Concern [6]. As of mid-December 2022, more than 82,000 Mpox cases have been reported globally, and more than 100 non-endemic countries—such as Colombia—have reported sustained transmission predominantly, though not exclusively, in men who have sex with men [3,7,8,9]. In Colombia, the first three cases of Mpox were reported on June 23 [10]. As of mid-December, more than 3900 laboratory-confirmed cases have been reported, most of them concentrated in two cities: Bogota (49.9%) and Antioquia (28.7%) [11]. At the time of writing this report, Colombia was the country with the second highest number of confirmed cases in South America, after Brazil [7,11].

Cartagena de Indias (10°25′25″ N, 75°31′31″ O) is one of the most touristic cities in Colombia and the Caribbean. The large and constant traffic of local and international travelers through the air and maritime routes makes Cartagena vulnerable to the introduction of various emerging infectious diseases. Here, we describe the epidemiological, clinical, and virological investigation of the first four cases of Mpox that were detected in Cartagena during the 2022 multi-country outbreak.

## 2. Materials and Methods

### 2.1. Case Definition and Identification

The cases described here were identified using passive surveillance conducted by local health authorities in Colombia and Cartagena, and all of them met the definition of a probable case of Mpox [11]. Lesion swabs, oropharyngeal swabs, and whole blood specimens from cases were collected and sent to Laboratorio de la Unidad de Investigación Molecular (UNIMOL) for testing. UNIMOL was the first laboratory in Colombia approved by the National Institute of Health (NIH) to test MPXV. 

### 2.2. Molecular Detection

Viral DNA was extracted individually from each sample using the TANBead Nucleic Acid Extraction Kit (Taiwan Advanced Nanotech; Ref.: W665A46) according to the manufacturer’s instructions. Then, eluates were used as DNA templates in real-time PCR assays using MPXV generic primers (G2RG) and strain-specific primers for clade II (G2RWA). Primers and PCR conditions were described elsewhere [11,12]. Then, from each case, the MPXV-positive DNA specimen with the lowest cycle threshold (Ct) value was selected for sequencing. 

### 2.3. Sequencing and Bioinformatics Processing

Next-generation sequencing (NGS) reads were generated in a MinION flow cell using a PCR-based amplicon approach with MPXV-specific primers [13,14]. Specifically, two multiplex PCRs per sample were performed by using previously described primers and PCR conditions, and clean-up steps and barcoding were performed with AMPure XP beads (A63882, Beckman Coulter, Brea, CA, USA) and with the Native Barcoding Expansion kit (EXP-NBD196, Oxford Nanopore Technologies, Oxford, UK), respectively [13,14]. NGS amplicon libraries were quantified by using the Qubit dsDNA High Sensitivity Assay kit (Q32854, Thermo Fisher Scientific, Waltham, MA, USA).

The basecalling was performed with guppy v.6.1.7 to convert the raw data to fastq files, and the basecalled files were then demultiplexed also with guppy v.6.1.7 [14]. Sequencing adapters were removed using Porechop v.0.2.4, low-depth sites (<10X) were masked with Ns, and preprocessed reads were mapped to an MPXV reference genome [14]. Consensus sequences were deposited in NCBI, and clades and lineages were determined using Nextclade (https://clades.nextstrain.org/, accessed on 3 November 2022). 

### 2.4. Viral Isolation and Plaque Assay

To assess infectiousness, all specimens were tested in Vero E6 cells using T12.5 cell culture flasks (Celltreat; Ref.: 229321) [15,16,17]. Briefly, on the infection day, the supernatant of confluent Vero E6 cells was discarded, and then cells were inoculated with 200 µL of a 1:10 dilution of the specimen in Dulbecco’s Modified Eagle Medium (DMEM; Sigma; Ref.: D6046) supplemented with 2% antibiotics (Sigma; Ref.: P4333). Flasks (Celltreat; Ref.: 229321) were incubated for 1 h at 37 °C in 5% CO_2_ and, every 15 min, were gently rocked manually back and forth five times. Later, 4 mL of DMEM supplemented with 2% fetal bovine serum (Sigma; Ref.: F4135) and 2% antibiotics were added. The cytopathic effect (CPE) was inspected daily, and 250 µL of supernatant was collected for testing MPXV DNA after the infection and when cells displayed >75% (3+) CPE. The isolation of MPXV was confirmed with PCR using generic primers in cell cultures [12,15]. Finally, to quantify infectious particles in the primary specimens, a solid plaque assay method was conducted in 24-well plates (Merck; Ref.: CLS3527) [15,18]. Plaque forming units (PFU) were counted for calculating PFU/mL.

## 3. Results

### 3.1. Case #1

A 37-year-old Colombian male residing in Cartagena, with no history of travel outside Colombia in the last 21 days. On 2 August, the subject sought medical attention for malaise, myalgia, and rectal pain with purulent discharge since 29 July, and for intermittent fever since 27 July. On the medical admission, the subject reported that on 30 July he had a maculopapular rash in the anorectal area and had been diagnosed with genital herpes and human immunodeficiency virus (HIV) infection approximately 5 years ago. The subject also reported having had sexual intercourse with a non-Colombian (unknown nationality) male on 24 July and having multiple sexual contacts in the last 21 days. Physical examination revealed numerous ulcers and verrucous lesions in the anal mucosa and perianal area. An anal lesion swab, oropharyngeal swab, and blood were collected for MPXV testing. 

### 3.2. Case #2

A 25-year-old Colombian male residing in Cartagena, with no recent travel history outside Colombia in the last 21 days. On 13 August, the subject requested medical attention via teleconsultation for having myalgia, fever, pruritus, and skin eruptions in various body areas. At the interview, the subject reported myalgia and fever since 31 July and vesicular-pustular rash in the genital area since 2 August, which later spread to the face and limbs. Also, the subject reported sexual intercourse with a non-Colombian individual (unknown nationality and date) and reported no multiple sexual contacts. No comorbidities were reported, and no physical examination was performed due to teleconsultation. A lesion swab from the genital area, an oropharyngeal swab, and blood were collected for MPXV investigation.

### 3.3. Case #3

A 32-year-old North American man arrived in Cartagena on 20 August. On 24 August, the subject sought medical attention due to a painless ulcer on the penis, malaise, asthenia, and swollen lymph nodes. At the interview, the subject reported fever, a single maculopapular lesion in his face since 19 August, and no comorbidities. In addition, the subject reported intercourse using condoms with his steady partner (unknown gender and nationality) on 16 August. The partner is HIV-positive and had skin lesions days after the intercourse. The physical examination revealed submaxillary cervical adenopathy and multiple maculopapular lesions on the upper lip of the mouth. A swab of the penile lesion, an oropharyngeal swab, and blood were collected for MPXV testing. 

### 3.4. Case #4

A 26-year-old Colombian male residing in Cartagena, with no history of traveling outside the country in the last 21 days. On 29 August, the subject sought medical attention for multiple genital vesicles, pruritus, malaise, arthralgia, myalgia, cough, odynophagia, and fever. The subject reported the onset of febrile illness and rash on 22 August and no comorbidities. Also, the subject reported having sex with men, multiple sexual contacts in the last 21 days, and intercourse on 8 August with a non-Colombian male (unknown nationality). Physical examination revealed numerous vesicles in the thorax. A genital vesicle swab and an oropharyngeal swab were collected. 

**Figure 1:**
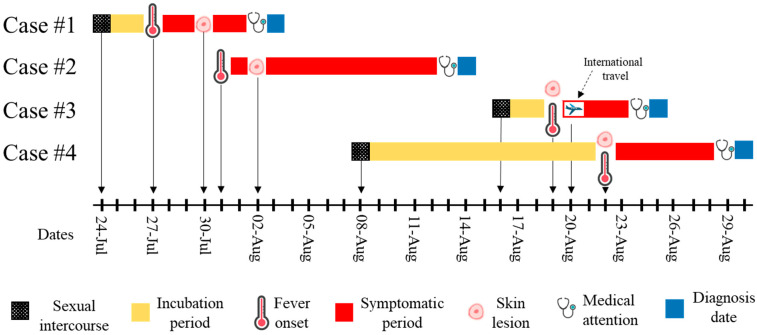
Timeline of the Monkeypox progression for the first four cases in Cartagena. The potential source of exposure, incubation and symptom periods, and date of the medical attention and diagnosis are represented using colors or figures. The timeline before medical attention was constructed using self-reported information and ends at the date of diagnosis.

The four cases had never received the smallpox vaccine, remained isolated at home, and received supportive care from their treating physician. Figure 1 displays a timeline describing the progression of Mpox, and Table 1 summarizes laboratory findings by case and specimen type. 

**Figure 2:**
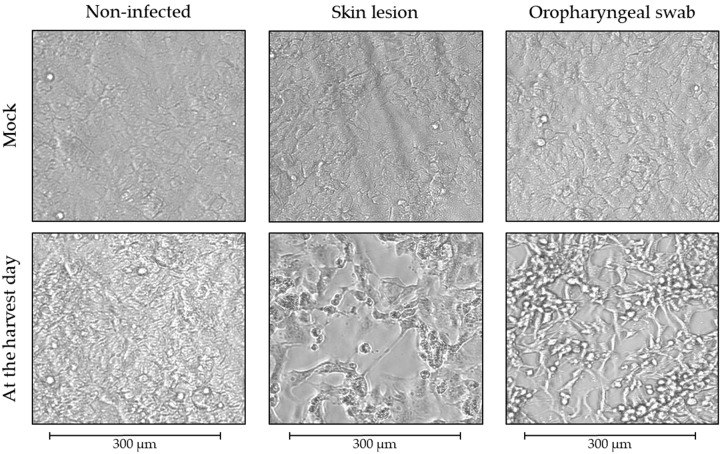
Cytopathic effect (CPE) observed in infected Vero E6 cells. On the harvest day, the cells displayed >75% (3+) CPE. The CPE observed in Vero E6 cells inoculated with the swabs collected from all cases is reported in Table 1. Cells inoculated with serum/plasma specimens showed no CPE and tested negative for MPXV on the harvest day. All mock non-infected cells tested negative for MPXV, and no CPE was observed during the follow-up. Images were captured before the infection and on the harvest day using the EVOS M5000 Imaging System (AMF5000, Thermo Fisher Scientific, Waltham, MA, USA).

Remarkably, all blood specimens tested negative using PCR and negative using viral isolation. Of the specimens evaluated per case, lesion swabs had the lowest Ct value, and, therefore, all were selected for sequencing. MPXV clade IIB and lineage B.1 were detected in all cases. Figure 2 shows the CPE observed on the harvest day in non-infected and infected Vero E6 cells. 

## 4. Discussion

As of mid-December, according to available official data, Cartagena was the city with the second highest number of Mpox cases in the Colombian Caribbean [11,19]. Notably, all reported cases were male (*n* = 15), and nearly of all of them were between 20 and 39 years of age (*n* = 14) [19]. In this study, we provide an in-depth description of the first four Mpox cases detected in Cartagena. Overall, the epidemiological, clinical, and virological features, including the laboratory findings described here, are consistent with characteristics described elsewhere during the 2022 global outbreak [8,9].

The conducted epidemiological investigation allowed us to identify the probable date of contact with a potential source of infection, and to describe the probable incubation period for all cases except for case #2. Given the debut with genital lesions and the reported sexual exposure, it is plausible to hypothesize that MPXV was transmitted through skin-to-skin sexual contact with an infected person [8,17,20]; however, the sexual contact with multiple partners hinders the accurate identification of the date and source of infection. As such, the incubation periods reported here may be biased since we have considered the report of the last sexual exposure as the source of infection and because of social desirability. However, the incubation periods presented in this report are consistent with those reported elsewhere [8,9,20,21]. Furthermore, based on the report of three apparently non-related cases (case #1, #2, and #4) with no history of recent travel and sexual contact with non-Colombian individuals, and the identification of one imported case (case #3), it is reasonable to suspect that multiple introduction events have occurred in Cartagena [3].

The occurrence of solitary genital lesions or multiple lesions in the genital and anal region, the later dissemination to other areas, and the symptoms reported by the four cases have been previously described [8,9,20]. The concomitant report of HIV and other sexually transmitted infections has also been previously described [8,9,22]. Interestingly, no complications such as secondary bacterial infections, proctitis, or tonsillitis, nor rare complications such as myocarditis [8,9], were identified among the cases described here, and no hospitalization or antiretroviral therapy was needed. However, it is important to note that the follow-up until diagnosis and/or a selection bias could explain the absence of complications and the no need for treatment observed here.

Herein, the Mpox diagnosis was confirmed with PCR using both lesion and oropharyngeal swabs. The viral isolation and the quantification of infectious particles were successful in all cases using lesion swabs, whereas, using oropharyngeal swabs, MPXV was isolated and quantified in two cases (cases #3 and #4). The high Ct value observed in the oropharyngeal swabs of cases #1 and #2 could correlate with a low viral load and thus explain the failure to isolate the virus [23]. In all the cases, the viral load (using the Ct value as a proxy) and the number of infectious particles were consistently higher in lesion swabs. Given our findings, it is plausible to hypothesize that cases could generate new chains of transmission mainly through close skin-to-skin contact [9,20,24]. Interestingly, the virus was not detected or isolated in any blood specimens tested. Laboratory findings described above are consistent with those reported elsewhere [8,9,15,16,23]; however, we consider that further research is required to understand the significance of having detected replication-competent particles in oropharyngeal swabs on ongoing and recommended prevention and control measures. 

Despite numerous reports worldwide, epidemiological and genomic information related to the 2022 multi-country outbreak remains scarce in South America [25]. This hinders our efforts to understand the molecular epidemiology of MPXV, both in Colombia and around the world. In this study, we report that viral characterization allowed the identification of MPXV clade IIB, which is related to the 2022 outbreak [26,27]. 

Although only four cases are described in this report, the comprehensive investigation provided useful information for health personnel and local health authorities. As of mid-December 2022, UNIMOL had tested more than 140 clinical specimens from multiple Colombian Caribbean cities, resulting in the detection of various Mpox cases in multiple departments of the Colombian Caribbean including Atlántico, Magdalena, Sucre, and Bolívar. To the best of our knowledge, this study constitutes one of the first reports that describe Mpox cases in the Colombian Caribbean region. In summary, our findings provide preliminary information that could potentially be useful for communicating effective prevention and control measures, and for alerting and strengthening the surveillance system at the local level.

## Figures and Tables

**Table 1:** Laboratory findings and viral characterization.

Case	Source of Infection ^a^	Skin Lesion Swab						Oropharyngeal Swab			
		G2RG/G2RWA ^b^	NCBI Accession Number	Genome Coverage/Average Depth	Day Post Infection/CPE ^c^	Day Post Infection/G2RG ^b^	PFU/mL	G2RG ^b^	Day Post Infection/CPE ^c^	Day Post Infection/G2RG ^b^	PFU/mL
#1	Unknown	21.5/18.2	OP390175	88.0%/315.7X	2/1+; 3/2+ 4/3+	0/18.5; 4/16.1	1.5 × 10^3^	38.6	6/1+; 7/2+; 8/3+	0/37.0; 8/Negative	0
#2	Unknown	19.1/17.0	Not deposited ^d^	53.3%/96.8X	7/1+ 8/2+ 9/3+	0/21.9; 8/30.8	1.0 × 10^1^	35.3	6/1+; 7/2+; 8/3+	0/36.5 8/Negative	0
#3	Imported	20.3/18.5	OP390179	88.8%/347.4X	1/1+; 2/2+; 3/3+	0/19.4; 1/24.6; 2/22.4; 3/17.3	6.0 × 10^3^	24.4	2/1+; 3/2+; 4/3+	0/24.4; 4/16.5	5.0 × 10^1^
#4	Unknown	22.2/21.9	OP390180	78.5%/247.6X	2/2+; 3/3+	0/21.8; 3/20.1	5.0 × 10^2^	28.4	6/1+; 7/2+; 8/3+	0/30.9; 8/31.7	3.0 × 10^1^

^a^ Classification based on the criteria described by the National Institute of Health of Colombia (https://www.ins.gov.co/Noticias/Paginas/Enfermedades-emergentes.aspx#hepatitis, accessed on 3 November 2022). ^b^ Cycle threshold (Ct) values are reported. ^c^ Days post infection with no cytopathic effect (CPE) are not reported. ^d^ Not deposited in the National Center for Biotechnology Information (NCBI) due to the low genome coverage.

## Data Availability

The data presented in this study are available on request from the corresponding author.
